# Supplementary material for: Characterization of cell-cell communication in autistic brains with single-cell transcriptomes
Source: J Neurodev Disord. 2022 May 2;14:29. doi: 10.1186/s11689-022-09441-1 (PMC9059394; doi:10.1186/s11689-022-09441-1)
Supplement: Supplementary file 3 — Additional file 3: Figure S1. Scatter plot for cell numbers in the 17 cell types and their numbers of CCC interactions. Figure S2. Heatmaps showing CCC differences between ASD and control PFCs in terms of counts (a) and strengths (b). The rows and columns indicate signaling sending and receiving cells, respectively. Figure S3. PCA plot based on pathway interaction strengths in ASD and control samples. Figure S4. Density plots for spatial expression correlation of actual L-R pairs identified in the scRNA-seq data or randomly paired L-Rs using either all spatial spots (a) or only spots in defined brain layers (b). Figure S5. Heatmaps showing CCC differences between ASD and control ACCs in terms of counts (a) and strengths (b). Figure S6. Pan cell type signaling networks identified in ASD ACC vs controls. Figure S7. Dot plots showing the change in relative contributions of each ACC cell type to outgoing (a) or incoming (b) signaling in ASD vs controls. Figure S8. Bubble plots showing the change in interaction strengths between individual pairs of cell types. The y-axis shows the differences computed with all cells, while the x-axis shows the differences computed with one half of the cells. In the latter analysis, 50% of the cells were randomly selected to run CellChat 10 times, the mean differences are plotted on the x-axis with the standard deviations from the 10 runs indicated by bubble sizes. [file 11689_2022_9441_MOESM3_ESM.pdf]

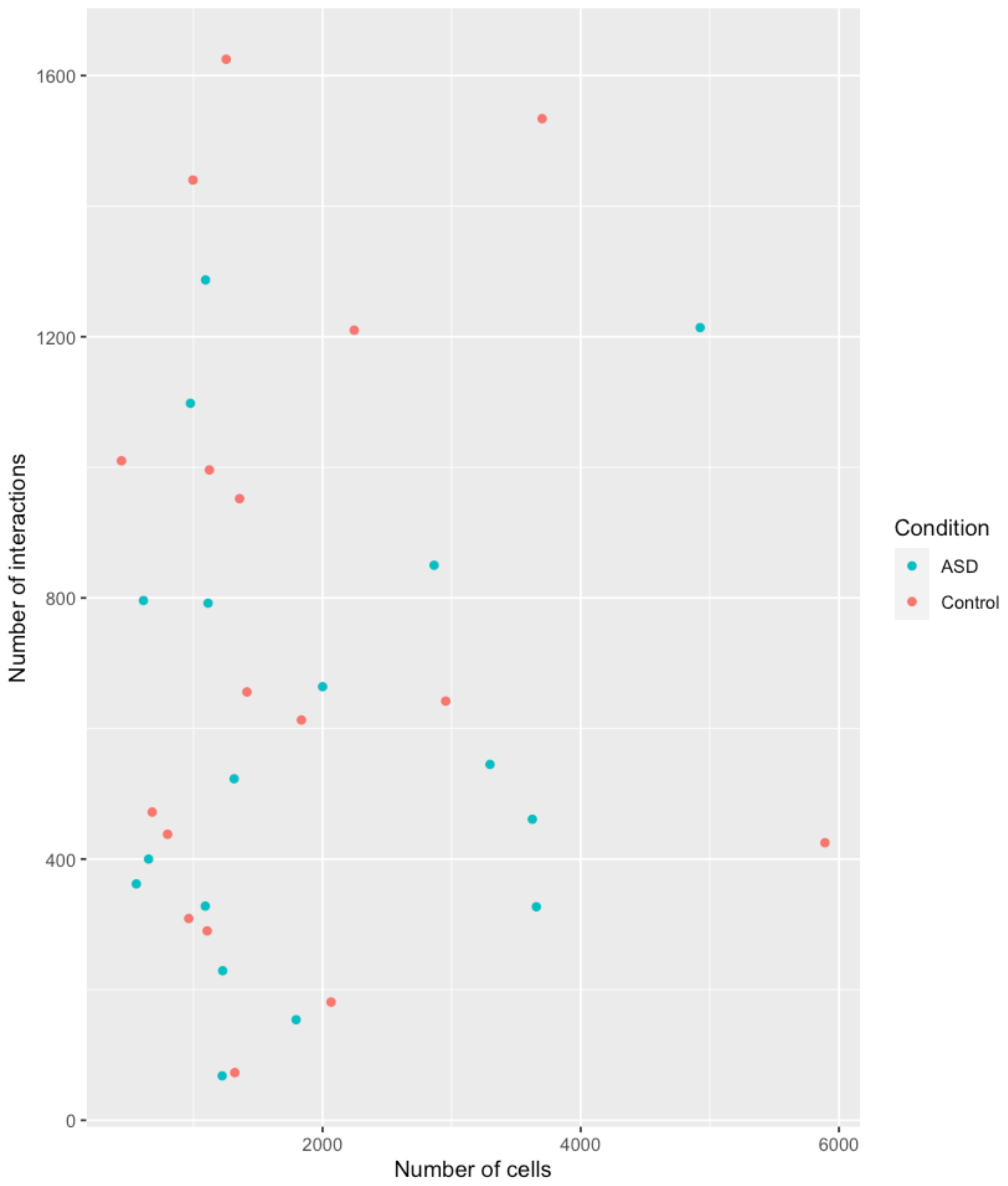

Figure S1. Scatter plot for cell numbers in the 17 cell types and their numbers of CCC interactions.

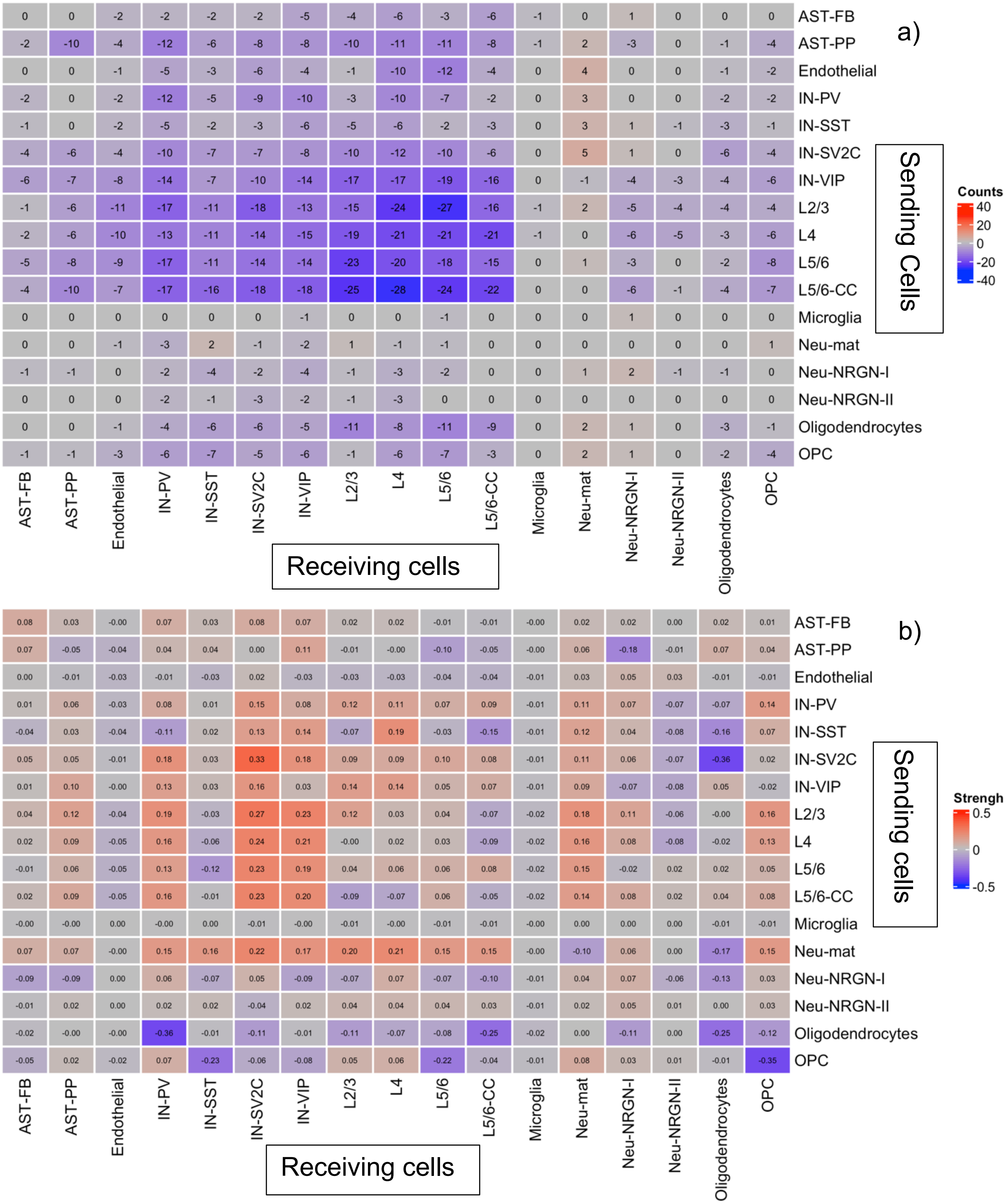

Figure S2. Heatmaps showing CCC differences between ASD and control PFCs in terms of counts (a) and strengths (b). The rows and columns indicate signaling sending and receiving cells, respectively.

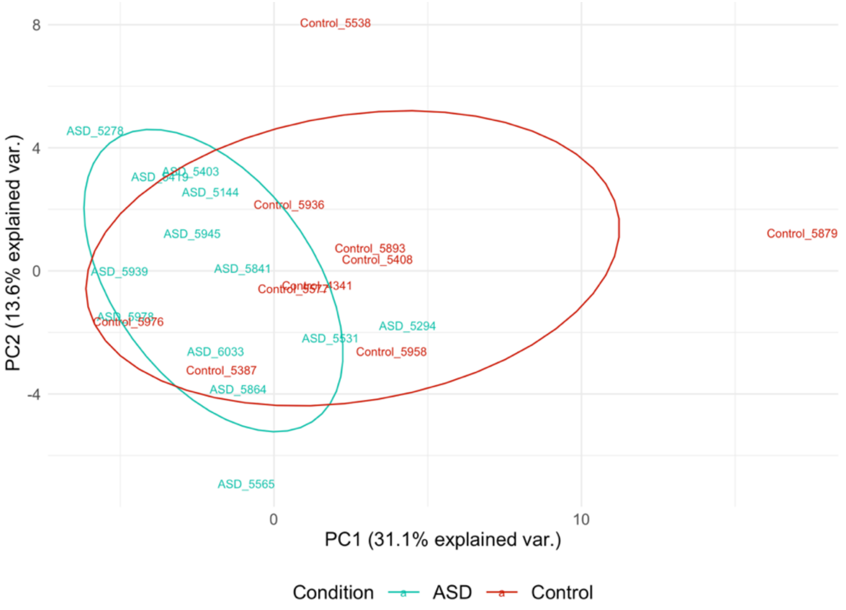

Figure S3. PCA plot based on pathway interaction strengths in ASD and control samples.

a)

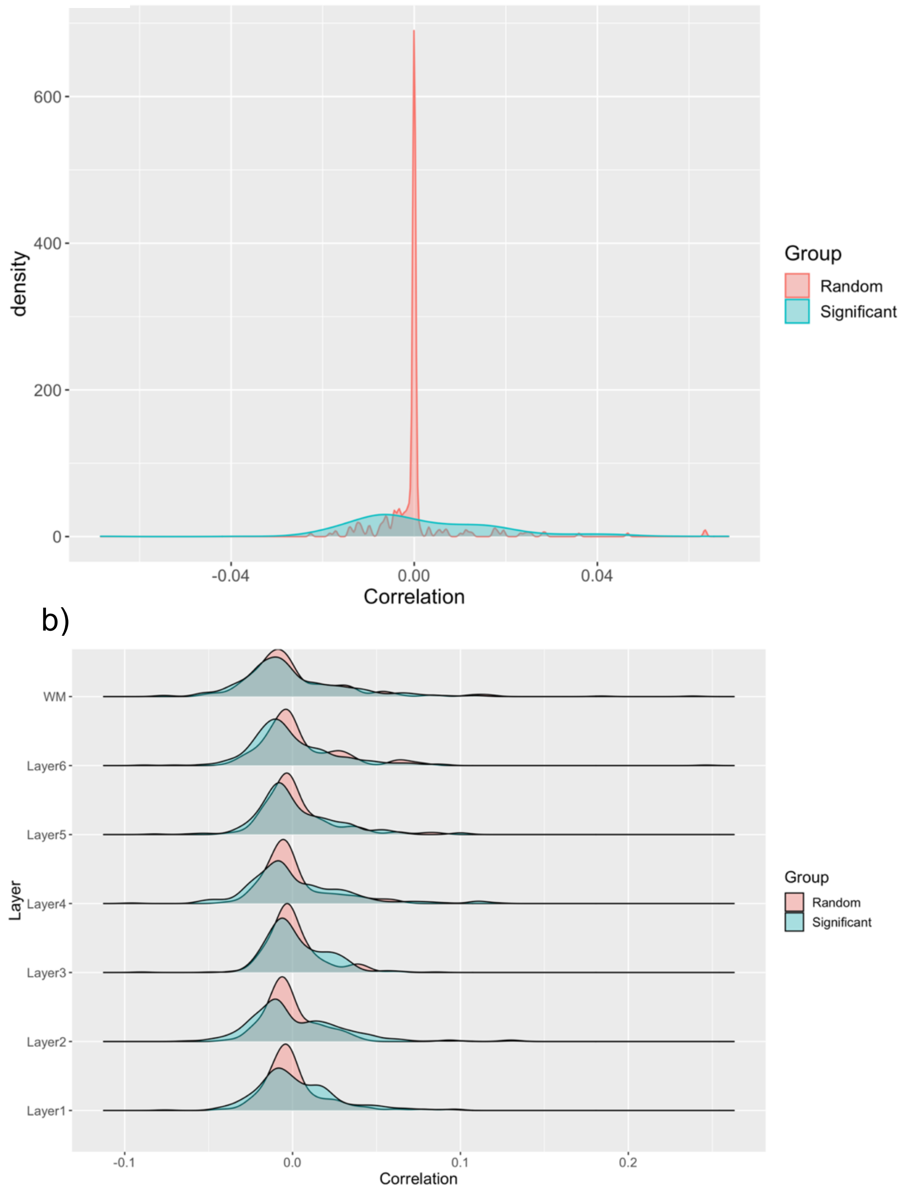

b)

Figure S4. Density plots for spatial expression correlation of actual L-R pairs identified in the snRNA-seq data or randomly paired L-Rs, using either all spatial spots (a) or only spots in defined brain layers (b).

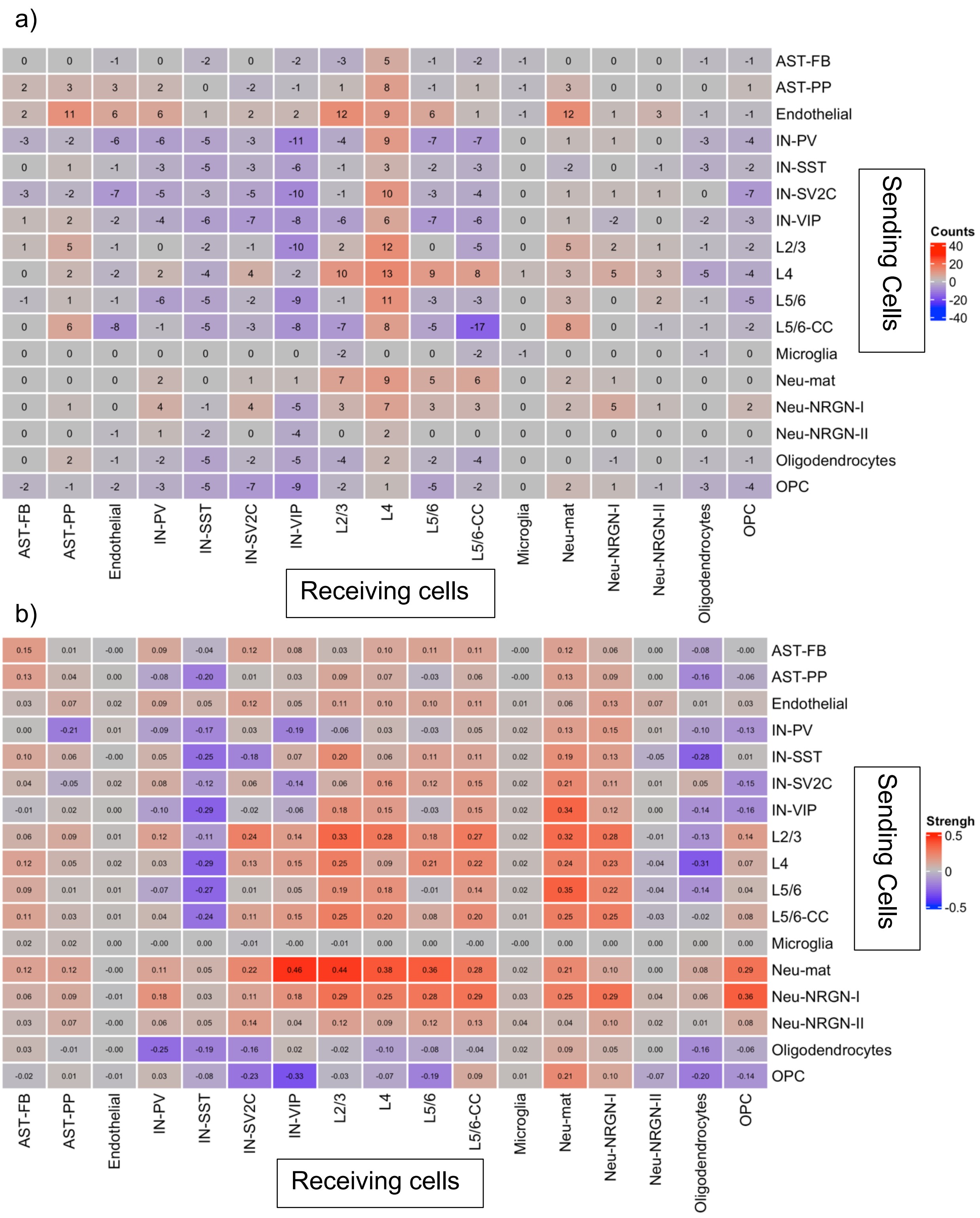

Figure S5. Heatmaps showing CCC differences between ASD and control ACCs in terms of counts (a) and strengths (b).

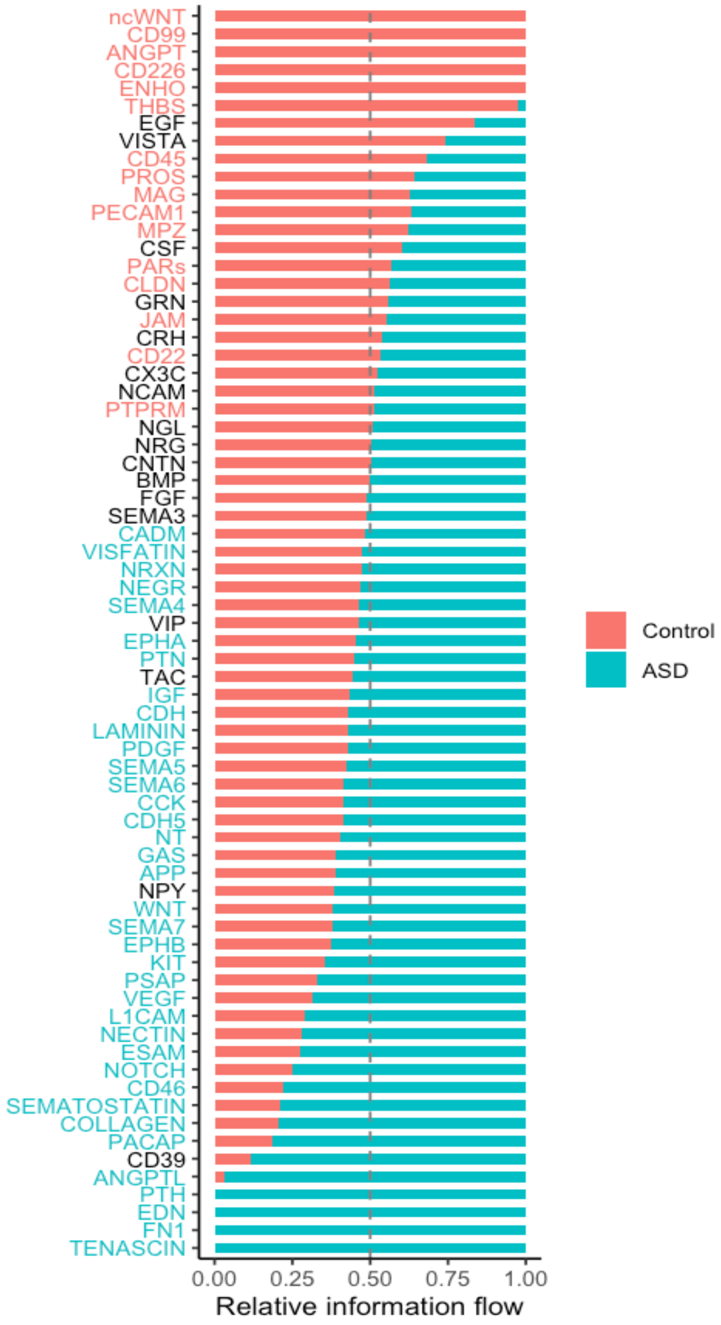

Figure S6. Pan cell-type signaling networks identified in ASD ACC vs controls.

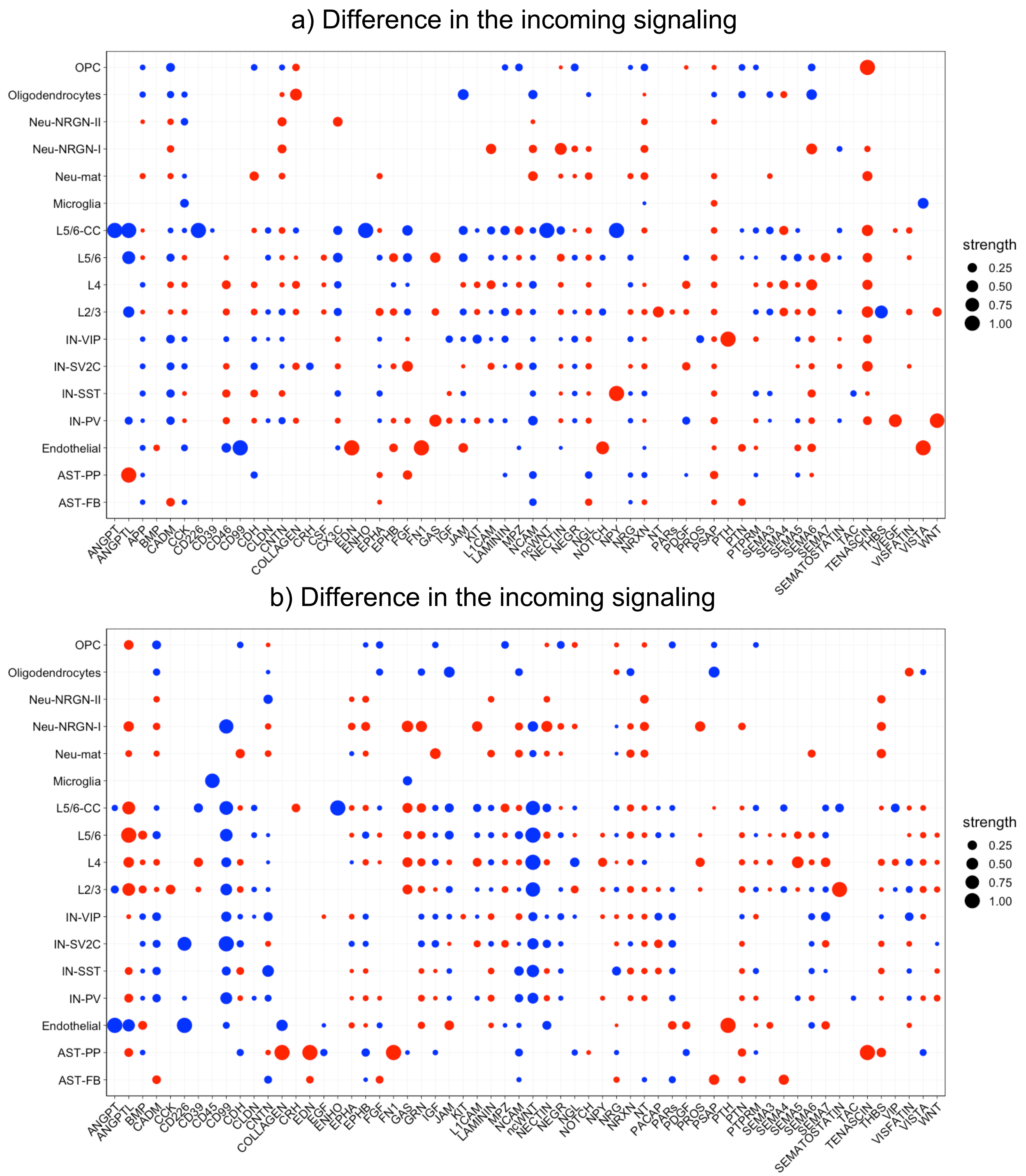

Figure S7. Dot plots showing the change in relative contributions of each ACC cell type to outgoing (a) or incoming (b) signaling in ASD vs controls.

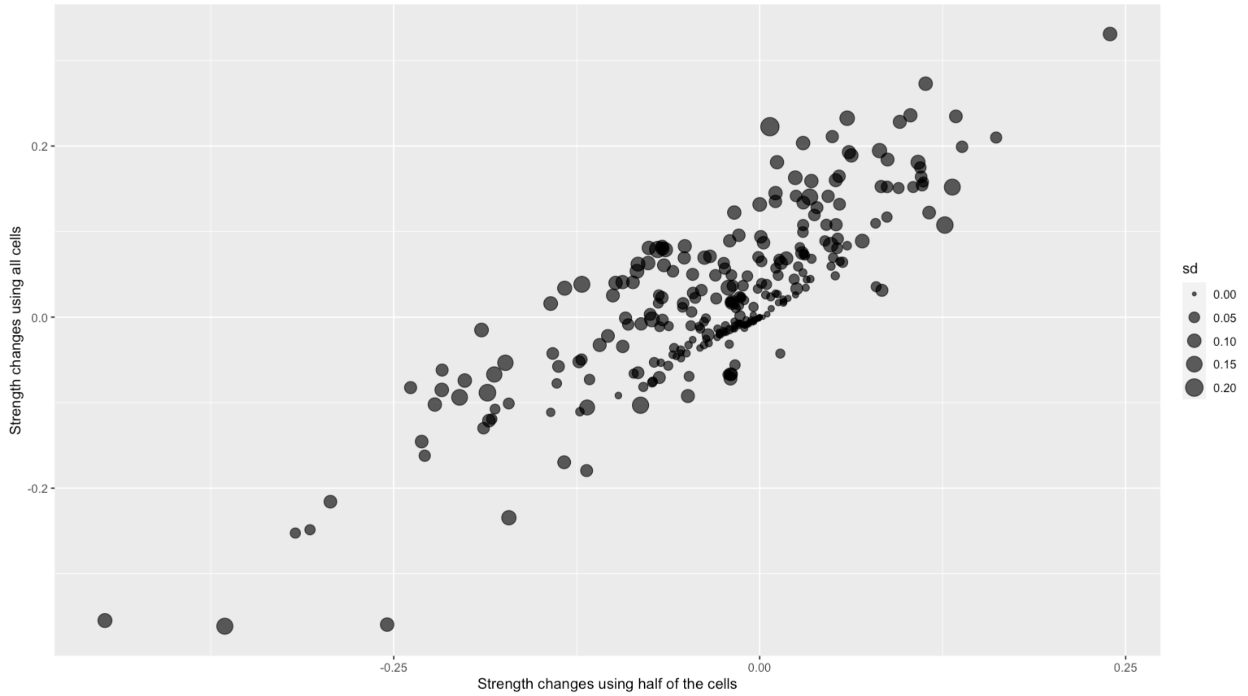

Figure S8. Bubble plots showing the change in interaction strengths between individual pairs of cell types. The y-axis shows the differences computed with all cells, while the x-axis shows the differences computed with one half of the cells. In the analysis of one half of the cells, 50% of the cells were randomly selected to run CellChat 10 times, the mean differences are plotted on the x-axis with the standard deviations from the 10 runs indicated by bubble sizes.
